# Can we use influencing factors to predict aspiration pneumonia in the United Kingdom?

**DOI:** 10.1186/2049-6958-8-39

**Published:** 2013-06-11

**Authors:** Judi Hibberd, Jenni Fraser, Claire Chapman, Hannah McQueen, Adrian Wilson

**Affiliations:** 1 Coventry and Warwickshire Partnership Trust, Nuneaton 18 Hodgetts Lane, Burton Green, Kenilworth, Warwickshire, CV8 1PJ, UK; 2 St Bartholomew’s Hospital, West Smithfield, London, EC1A 7BE, UK; 3 Adult Speech and Language Therapy Service, Marriott Ward, Victoria Building, Leicester Royal Infirmary, Leicester, UK; 4 Department of Clinical Physics & Bioengineering, University Hospital Coventry, Coventry, UK; 5 Warwickshire NHS Trust, University Hospital Coventry and Warwickshire, Coventry, UK

**Keywords:** Aspiration pneumonia, Dysphagia, Influencing factors

## Abstract

**Background:**

The current study builds upon the work of others in looking at influencing factors of aspiration pneumonia in people with a swallowing problem. This study differs from previous researches on this topic, focusing on the United Kingdom (UK) population and involving more recently defined influencing factors of aspiration pneumonia. The study aims to explore the multifactorial nature of aspiration pneumonia in a UKdysphagic client group, as well as different disease specific variables.

**Methods:**

Speech and Language Therapists collected data on 33 influencing factors over a period of 6 months during routine bedside swallowing assessment of 687 patients. All subjects were adults referred with suspected dysphagia and included acute inpatients, head and neck cancer patients and adults with learning disabilities. The study population included 400 males and 287 females and ages ranged from 17 to 102 giving a mean age of 72.9 years. The influence of the different variables included in the study was evaluated using multivariate logistic regression analysis.

**Results:**

The results show that 13 statistically significant influencing factors were implicated in the development of aspiration pneumonia for this group. Out of these, nine correlate with the previous work undertaken in the United States. These were poor mobility, nil by mouth status, age, dependency for feeding, number of medications, Chronic Obstructive Pulmonary Disease (COPD), number of medical conditions, stroke and alcohol abuse. Four further influencing factors were shown to be significant in the UK population, these were dysphagia, only oral intake, bedfast, and male gender.

**Conclusions:**

This study confirms that in the UK there are influencing factors in the development of an aspiration pneumonia. It would be prudent to remember that a direct link is yet to be established when applying this knowledge to inform clinical management.

## Background

The assumption that aspiration pneumonia is a condition caused as a direct result of aspiration of foods or fluids has been extensively studied in recent years [1-3]. In the field of dysphagia management, Speech and Language Therapists assess and advise on the safety of clients’ swallowing, by determining the likelihood of aspiration occurring. Conventional wisdom continues to hold that aspiration of any material into the lungs can lead to aspiration pneumonia and therefore any sign of aspiration is currently managed conservatively [1].

As aspiration pneumonia has been reported as the 5^th^ leading cause of death in the United States and the 4^th^ most frequent cause of death in the elderly [4], research has begun to analyze what causes aspiration pneumonia and how we can prevent it.

Speech and Language Therapists, supported by a growing body of research [3-6] have noted that not all patients who aspirate develop aspiration pneumonia. A number of studies including those by Langmore et al., [2], Marik [5] and Schindler et al. [7] found several significant influencing factors for developing aspiration pneumonia highlighting that aspiration pneumonia is a multifactorial condition and thus demonstrating that aspiration is necessary, but not solely sufficient to cause an aspiration pneumonia. Combined together, this strongly questions the assumption that aspiration pneumonia is a direct and inevitable consequence of aspiration.

As a result of the assumption that aspiration is causally linked to aspiration pneumonia, in the UK patients with aspiration are currently managed using modified oral intake or recommended to be nil by mouth. If it is a misconception that aspiration inevitably leads to aspiration pneumonia, then it would be difficult to defend the continued practice of assigning to alternative/non-oral management regimes that are detrimental to their quality of life whilst also being costly, invasive and distressing [1,4,6-9] and many patients with swallowing problems could continue to eat and drink orally.

For the purposes of this study, the influencing factors for aspiration pneumonia suggested in the literature have been grouped into the following categories: oral health and hygiene, feeding, ability and issues, immune system depression, medical conditions (including medication), consciousness level, mobility and a category for other factors not covered above.

Several studies have identified oral health and hygiene as influencing factors associated with aspiration pneumonia [2,7,10-13]. Pace and McCullough [10] suggest that when the normal aetiology of the swallow is compromised, this affects how the oropharyngeal flora itself grows and manages pathogens, which can then precipitate aspiration pneumonia. It is interesting to note that some sources are suggesting that non-oral feeding techniques such as Naso gastric feeding tube(NG) tubes can also negatively impact on a patient’s ability to avoid aspiration itself [7,13] and on oral health, which can lead to aspiration and aspiration pneumonia [13]. Langdon et. al [12] note that patients who were fed orally after a stroke were significantly less likely to develop a respiratory infection than those who were ‘nil-by-mouth’ due to the continued aspiration of saliva and reflux; both of which are positively associated with pneumonia. In addition reflux as a result of poor NG tube placement has been associated with aspiration [7,12,13]. This suggests that traditional methods to manage the risk of aspiration pneumonia are not only used too frequently but are not necessarily effective and could pose additional risks for patients.

Some studies to date [5,14] suggest that feeding issues such as dependency for feeding are linked to impaired cognition and mobility in the patient which can influence the development of aspiration pneumonia. Furthermore, an untrained or inexperienced carer can have a negative impact on feeding effectively if intake is given too quickly or in quantities that are too large to be swallowed safely. This can lead to aspiration despite the risk management technique of assisted feeding having been advised [3,14].

Some studies indicate that immunosuppression as a result of either previous medical conditions, surgery or lifestyle choices impact negatively on the individual’s ability to fight infection and this puts them at risk of aspiration pneumonia. Schindler and Bouchard [7,15] report that individuals with malnutrition are at risk of aspiration pneumonia whilst Marik and Kaplan [16] report that decreased immune system activity as a result of aging may be an influencing factor in aspiration pneumonia. Lifestyle factors such as heavy alcohol consumption [17,18] and smoking [19] may also impact on an individual’s ability to fight infection and thus increase the risk of aspiration pneumonia.

Medical conditions have a demonstrated link to immunosuppression as discussed above and also to decreased mobility and dependence for feeding. Key to this influencing factor is the link between the medical conditions and disruption of the normal co-ordination of feeding and swallowing and the effect of some medications on the oropharyngeal system. Stroud et al. [20] note that any issue with any area involved in the normal swallow process, such as tracheostomy, can lead to an increased risk of aspiration and aspiration pneumonia. Chronic Obstructive Pulmonary Disease (COPD), [6,21] Congestive Heart Failure (CHF), [22], Stroke [23] (which itself can predispose to aspiration) and Parkinson’s Disease [15] have all been suggested to impair the co-ordination of breathing and, in turn, impact on swallowing ability, thus increasing risk of aspiration and aspiration pneumonia. Cancer has also been associated with aspiration and aspiration pneumonia in that it relates to immune suppression, complications with the pulmonary and respiratory systems and decreased ability to cough and clear secretions [24].

Gallagher and Naidoo [25] suggest that xerostomia (dry mouth) is a well known adverse effect of many medications. Xerostomia impacts on oral health and hygiene and the individual’s ability to clear the mouth of bacteria. This increases the risk of aspirating altered pathogens which may, in turn, precipitate aspiration pneumonia [16]. All these medical conditions and medications affect the normal swallow and normal oropharyngeal functioning, thus decreasing the individual’s ability to fight off infection which further compromises the immune system and so increases the risk of aspiration pneumonia.

Stroud et al. [20] note ‘reduced levels of consciousness’ compromises the cough reflex and the normal swallow process. Thus patients with a reduced level of consciousness may be predisposed to aspirate and have a reduced ability to clear aspirated material from the lungs.

Loeb et al. [26] associated the lack of mobility with increased risk of aspiration pneumonia, as did Langmore [2]. The suggestion is that inability to move around inhibits the ability to clear secretions and to clear the lungs: an effect which is observed to increase with age.

Although some studies have identified the same influencing factors for aspiration pneumonia, they have attributed varying significance to those discussed factors. Marik and Kaplan [16] for example concluded that dysphagia maybe a ‘significant predictor’ of aspiration pneumonia, whilst Langmore [2,27] rated it as one, but not the strongest of its 18 significant predictors. Whilst there are clear benefits to optimizing the feeding regimes of patients who aspirate, there appears to be no consistent pattern of the influencing factors within these studies, to enable us to predict those patients that will go on to develop aspiration pneumonia. In order to use this research to safely inform our management, we need to be able to accurately identify those patients who are at risk of aspiration pneumonia and those who are not and it appears it is the relative importance of each influencing factor in the development of aspiration pneumonia, which needs to be better understood. Already in clinical use there are various water protocols, which are supporting the development of this understanding, allowing patients who are at risk of aspiration to take fluids orally by incorporating and managing some of these influencing factors [1,3,4].

Other factors found to influence the development of an aspiration pneumonia are age and gender, both reported by Langmore [2].

The majority of researches in this area has been conducted in residential care settings (care homes), rehabilitation units and hospital environments in the USA, a different population to that of the acute and community settings of the UK and thus the findings from that research may not be applicable to other patients groups – in particular to the patients groups managed by speech and language therapists in the UK. The aim of this study was to identify whether the influencing factors identified in the research and others commonly collected by UK Speech and Language Therapists [28] were influential in the development of aspiration pneumonia in the UK population and could thereby enable a change in our patient management.

## Methods

Data were collected on 687 consecutive patients referred to the adult speech and language therapists with suspected dysphagia. These included acute inpatients, head and neck cancer patients (both inpatients and outpatients) and adults with learning disabilities (both inpatients and outpatients). A current aspiration pneumonia was recorded if a medical diagnosis had been made or confirmed and documented in the patient’s medical notes by the patient’s consultant physician. The data items processed were those which formed part of, or were derived from, the standard set of data collected by Speech and Language Therapists in the Midlands in the UK [26]. The data set for analysis (Table 1) consisted of 26 potentially influencing factors of which 1 was continuous (age), 2 were interval (number of medical conditions and number of medications) and the remainder were categorical.

**Table 1:** Subject demographics

	**Adult learning disability**		**Head and neck cancer**		**Other**	
	**Non aspiration pneumonia**	**Aspiration pneumonia**	**Non aspiration pneumonia**	**Aspiration pneumonia**	**Non aspiration pneumonia**	**Aspiration pneumonia**
Number of subjects (M/F)	8/8	3/6	23/16	1/3	269/211	96/43
Age in years (mean ± sd)	52 ± 17	56 ± 11	59 ± 13	70 ± 17	73 ± 16	80 ± 13

Age and number of subjects as a function of the source of referral.

The study protocol and data collection methods were approved by the medical ethical committee of University Hospital Coventry and Warwickshire.

In the first part of the analysis, a univariate analysis was performed to identify which of the potentially influencing factors was statistically and significantly different between subjects who were diagnosed with an aspiration pneumonia and those who were not. For the categorical variables a *χ*^2^ test was used whilst for the interval and continuous variables the Student’s *t*-test with pooled variance was used.

In the study subjects were classified on referral to the Speech and Language Therapy service as being: adults with learning disabilities; diagnosed with a head and neck cancer; or other. To determine whether the first one of two groups affected the factors associated with aspiration pneumonia, the univariate analysis was also run with the 619 subjects with the referral diagnosis recorded as ‘other’.

To ensure the results of this study are comparable with those from other workers who have tried to identify influencing factors for Aspiration Pneumonia their approach of not correcting probability levels for multiple comparisons has been followed [2,5].

The univariate analysis determines whether there are statistically significant group differences in the variables between subjects with and without an aspiration pneumonia. However, a predictor based on the measures collected which could identify subjects likely to develop aspiration pneumonia in the clinical environment would be valuable. As a first stage in determining whether such a predictor is possible a series of logistic regression analyses were performed on those measures for which a univariate statistical significance of p < 0.05 was obtained. The logistic regression can include both categorical and continuous variables and a backward elimination technique was used to ensure that the resultant models included a minimum number of terms (variables). The logistic regression analysis was performed using SPSS (IBM Inc.) version 19.

## Results

Table 1 gives the age and sex details of the subjects in the study. This table shows that the majority of the subjects in the study were classified under the ‘Other’ heading, and that the majority of whom were adult acute inpatients. It should be noted the number of subjects in the group’s adults with learning disabilities and head and neck cancer was small and an analysis of these was not possible.

Table 2 shows the 26 influencing factors on which statistical analysis was performed arranged in descending order of the probability that they show a group difference between subjects who were and who were not diagnosed with an aspiration pneumonia. Using a cut-off level of p <0.05 shows that 13 factors have a statistically significant group difference – 8 categorical, 2 interval and 1 continuous.

**Table 2:** The 26 influencing factors

	**Factor**	**Number/value in aspiration pneumonia**	**Number/value in Non-aspiration pneumonia**	**P**
		**n = 152**	**n = 535**	
1	Mixed tube and oral feeding	56	105	**0.000**
2	Poor mobility	6	100	**0.000**
3	Age in Years (mean ± sd)	78 ± 14	71 ± 17	**0.000**
4	Dependency for feeding	102	287	**0.004**
5	Only oral feeding	59	274	**0.009**
6	Dysphagia	121	366	**0.010**
7	Number of medical conditions (mean ± sd)	3.3 ± 1.8	2.8 ± 1.8	**0.011**
8	Bedfast	63	161	**0.011**
9	COPD	19	33	**0.015**
10	Number of medications (mean ± sd)	6.6 ± 3.2	5.8 ± 3.5	**0.016**
11	CVA	48	229	**0.017**
12	Alcohol abuse	12	83	**0.023**
13	Male	52	235	**0.040**
14	GI disease	62	178	0.105
15	Dental disease	39	104	0.120
16	Chronic Heart Failure	19	43	0.125
17	Any feeding tube	37	156	0.287
18	Good oral care	87	331	0.348
19	Smoker	15	70	0.356
20	Mechanically altered diet	30	88	0.408
21	Moves with assistance	83	274	0.518
22	Cancer	23	73	0.738
23	Weight loss	18	57	0.789
24	Dry mouth	21	80	0.826
25	Medications causing a dry mouth	102	355	0.940
26	Tracheostomy	7	22	0.969

*COPD* Chronic obstructive pulmonary disease, *CVA* Cerebral vascular accident.

The 26 influencing factors used in the analysis together with the statistical probability of a group difference. The number of cases in each category is given for the categorical variables and the mean ± sd for the continuous and interval variables.

The univariate analysis of the 619 subjects with a referral of ‘Other’ yielded the same influencing factors in the same order of significance with the exception of the final factor, ‘Alcohol Abuse’ which failed to reach statistical significance.

In the logistic regression analysis, all the 13 variables were input to the logistic regression analysis and the best detection of aspiration pneumonia was achieved with 12 of the 13 variables plus a constant. The only variable not included in the model was ‘Only Oral Feeding’. However the model only correctly identified 25 of the 152 subjects (16%) diagnosed with an aspiration pneumonia. Conversely, 525 out of the 535 subjects who were not diagnosed with an aspirate pneumonia were correctly classified (98%).

## Discussion

The findings of this study support the suggestion in the literature that there are influencing factors in the development of an aspiration pneumonia. It identifies 13 statistically significant influencing factors that may predict aspiration pneumonia in the UK from an original list of 26.

As discussed previously, for the purpose of this study, the influencing factors for aspiration pneumonia suggested in the literature were grouped into the following categories: oral health and hygiene, feeding ability and issues, immune system depression, medical conditions (including medication), consciousness level and mobility and other influencing factors.

From the category of oral health and hygiene the strongest influencing factor of mixed tube and oral feeding came. This was a derived influencing factor due to a limited sample size for individual tube feeding and also the need to take into account those patients who were tube fed and took some oral intake which may have been at risk of aspiration. As this was a derived influencing factor it is difficult to say whether it was either the tube feeding or oral feeding or indeed both which gave rise to the statistical significance. Previous studies by Schindler and Logson [7,13] highlighted that NG feeding can negatively impact on a patient’s ability to avoid aspiration. It could be suggested that this data supports their findings.

The influencing factors of gastro-intestinal disease, dental disease, only tube feeding and good oral care did not reach significance for this category. Although Langmore and Pace [2,10] have found poor oral care to be directly related to the acquisition of aspiration pneumonia, our data collection focused on good oral care. It might be suggested that as the influencing factor of good oral care did not reach significance, the influencing factor of poor oral care may still be implicated.

In the category of mobility, the influencing factors of poor mobility and bedfast were significant. Poor mobility and bedfast were collected using an adapted tool based on Langmore’s study [2]. This outcome replicates previous findings from Loeb et al. [26] and Langmore [2] who noted that a lack of mobility increased the risk of aspiration pneumonia. Data on assisted moving was also collected but were not found to be a significant influencing factor. The more mobile a patient is the less likely he is to be at risk of developing aspiration pneumonia.

In the category of other influencing factors, age and male gender were noted to be significant. Langmore’s study [2] reported increased incidences of aspiration pneumonia with advanced age which they attributed to reduced immunity and increased comorbidities. This study produced results which corroborate this point, suggesting the possibility that this could also be true of the UK population.

Male gender was highlighted as a potentially new influencing factor whereas Langmore [2] highlighted female gender as an influencing factor in her study. A possible explanation for this may be that females were more prevalent in Langmore’s study, whereas in our study males were more prevalent.

The category of feeding ability and issues yielded three statistically significant influencing factors: dependency for feeding, only oral feeding and dysphagia.

Carlaw [3] and Siebens [14] both suggested that dependency for feeding is linked to impaired cognition and poor mobility and that an untrained or inexperienced carer can have a negative impact on effective feeding. Our results would support this finding.

Only oral feeding is also a potentially new influencing factor. This factor was derived from data collection concerning presence of a feeding tube, mixed tube and oral feeding or only oral feeding. The significance of this influencing factor may be due to the sample size of the data set with only oral feeding being the largest in number. However this could also be linked to aspiration pneumonia being multifactorial in nature.

Dysphagia alone as an influencing factor in the development of an aspiration pneumonia is a contentious issue and our findings are consistent with Marik’s study [16] which showed that dysphagia is an influencing factor. However like Langmore [27], the results of this study would suggest that dysphagia on its own is not enough and that other influencing factors need to be present for the development of an aspirate pneumonia.

In our study, having a mechanically altered diet in the category of feeding ability and issues did not reach significance unlike Langmore’s [2] and Siebens’ [14] studies. This may be explained by the necessity for our patients on mechanically altered diets to feed themselves, thereby reducing the negative impact of dependency for feeding.

Immune system depression yielded two statistically significant influencing factors, that is number of medications and alcohol abuse, as well as four un-statistically significant influencing factors, that is smoking, weight loss, dry mouth and medications causing a dry mouth.

Previous studies [2,25] have reported that the increased number of medications reduce salivary flow and so predispose the growth of bacteria that may then be aspirated. The number may also indicate that a person has multiple comorbidities and therefore a compromised immune system.

Unlike other studies, our results have indicated the number of medications that are implicated in developing aspiration pneumonia. Our study gave a number of 6 and above as being statistically significant.

The univariate analysis of the 619 subjects with the referral diagnosis of Other’s yielded the same influencing factors in the same order of significance with the exception of the final factor,’ Alcohol Abuse’ which failed to achieve statistical significance.

Alcohol abuse was found to be a significant influencing factor by Happel [18] and Gluckman [17] who suggested that in alcohol abuse, host defences are reduced leading to the development of aspiration pneumonia and our study would support these findings.

Gonzalez [19] reported that smoking increased the chances of aspiration pneumonia; however our study did not corroborate this, perhaps due to having a small sample size within this data set.

Bouchard [15] suggested weight loss was an influencing factor, but our results did not support this issue. Data about weight loss, as described by Bouchard, were collected from the medical notes. It is possible that this documentation lacked the detail required to ensure these data were collected appropriately. A further study with more focus on this link is therefore suggested.

Dry mouth was assessed using a visual assessment formulated from a local oral assessment tool. Medications causing a dry mouth were checked with the British National Formulary.

In contrast with other studies [25] we did not find a correlation between dry mouth and medications causing a dry mouth and the development of aspiration pneumonia. This is possibly due to the effective way our services manage dry mouths in patients.

There is a strong body of research around the prevalence of different medical conditions and the development of aspiration pneumonia [6,21,22,24]. Our study highlighted that COPD and/or Cerebral vascular accident (CVA) were significant influencing factors. Coyle [21] suggested COPD impairs the co-ordination of breathing and in turn impacts on swallowing ability, thus increasing risk of aspiration and aspiration pneumonia. Holas [23] found a direct correlation between CVA and the development of aspiration pneumonia. Our study would agree with both these findings. Unlike other studies we have identified a number of 3 or more medical conditions to be statistically significant in the development of an aspiration pneumonia. This would concur with the assumption that the more medical conditions a patient has the more likely his immune system is to be compromised.

The influencing factors of Congestive heart failure (CHF) and cancer were not found to be statistically significant in our study, unlike other studies [2,24]. This may again be due to the limited data set for these groups in our study.

The final category was conscious level. Initially this category included reduced level of consciousness to reflect the literature found. However, in clinical practice, we were not assessing patients if they were not deemed sufficiently alert and therefore we found we did not have any subjects for analysis.

Tracheostomy was included in this category. Unlike Stroud [20] we found tracheostomy was not statistically significant in our data set, which again may reflect the small data set for this group of patients in our study.

Although this study looked at 26 factors there may be other factors which need to be included in any further studies. The sample sizes in some of the influencing factor data sets were small and this can surely be considered a limitation of this study.

It is possible that extending the length of the study would have increased the number of participants in the data sets and thereby negated the need for some of the derived variables.

Some of the influencing factors such as good oral care relied on subjective testing. The use of a specific tool to ensure objectivity and replicability of data would be advised in future studies.

Our study has been based upon the populations of acute and community patients in the UK rather than the nursing home residents of previous studies, and it has become clear that the development of an aspiration pneumonia in this population is multifactorial in nature. Although this study has produced a statistically significant list of influencing factors in the UK population, it was not possible using a logistic regression analysis to predict which of the subjects had an aspirate pneumonia from these factors. Perhaps more importantly it was not possible to identify which of the influencing factors need to be present for subjects to go on to develop an aspiration pneumonia. Further work is therefore needed to define that relationship between the factors and to look at disease specific relationships. More research is also required before the association between these factors and their influence on aspiration pneumonia is also clearly defined in the UK population.

## Conclusions

This study confirms that in the United Kingdom there are influencing factors in the development of an aspiration pneumonia and that it is multifactorial in nature. A direct link between individual factors and groups of factors and aspiration pneumonia are to be established yet.

Further research is needed before we can use our knowledge of these influencing factors to inform our clinical management of dysphagia. We look forward to the development of a validated tool for use in clinical practice.

## Abbreviations

CHF: Congestive heart failure; COPD: Chronic obstructive pulmonary disease; CVA: Cerebral vascular accident; NG: Naso gastric feeding tube; UK: United Kingdom.

## Competing interests

None of the authors has any competing interests.

## Authors’ contributions

JH conceived the study, participated in the design of the study and coordination and helped to draft the manuscript. JF conceived the study, participated in the design of the study and coordination and helped to draft the manuscript. CC participated in the design of the study and helped to draft the manuscript. HM participated in the design of the study and helped to draft the manuscript. AW performed the statistical analysis and helped to draft the manuscript. All authors read and approved the final manuscript.

## Authors’ information

JH

Speech and Language Therapist working solely with dysphagia, in adults and adults with learning disability and paediatrics. Previous joint author of article written about the development of aspiration pneumonia and dysphagia. Royal College of Speech and Language Therapist advisor, for dysphagia. Fellow of the Royal College of Speech and Language Therapists, for Clinical Excellence 2012.

JF

Consultant Speech and Language Therapist for Adults with Learning Disability.

Previous joint author of article written about the development of aspiration pneumonia and dysphagia.

CC

Specialist Speech and Language Therapist in Voice and Head&Neck cancer.

With colleagues she is currently reviewing the literature available for floor of mouth surgery in oral cancer patients and the effects on swallowing, aiming to develop a research project based on our findings.

HM

Clinical Lead Speech and Language Therapist for Stroke. Currently working towards a masters degree.

AW

Director of Clinical Physics and Bioengineering at large acute hospital and Professor of Medical Physics at University of Warwick.

Author of over 100 peer reviewed articles and reports.
